# An Assessment of the Breastfeeding Practices and Infant Feeding Pattern among Mothers in Mauritius

**DOI:** 10.1155/2013/243852

**Published:** 2013-06-24

**Authors:** Ashmika Motee, Deerajen Ramasawmy, Prity Pugo-Gunsam, Rajesh Jeewon

**Affiliations:** ^1^Department of Health Science, Faculty of Science, University of Mauritius, Reduit, Mauritius; ^2^Faculty of Law and Management, University of Mauritius, Reduit, Mauritius; ^3^Department of Bioscience, Faculty of Science, University of Mauritius, Reduit, Mauritius

## Abstract

Proper breastfeeding practices are effective ways for reducing childhood morbidity and mortality. While many mothers understand the importance of breastfeeding, others are less knowledgeable on the benefits of breastfeeding and weaning. The aim in here is to assess breastfeeding pattern, infant formula feeding pattern, and weaning introduction in Mauritius and to investigate the factors that influence infant nutrition. 500 mothers were interviewed using a questionnaire which was designed to elicit information on infant feeding practices. Statistical analyses were done using SPSS (version 13.0), whereby chi-square tests were used to evaluate relationships between different selected variables. The prevalence of breastfeeding practice in Mauritius has risen from 72% in 1991 to 93.4% as found in this study, while only 17.9% breastfed their children exclusively for the first 6 months, and the mean duration of EBF (exclusive breastfeeding) is 2.10 months. Complementary feeding was more commonly initiated around 4–6 months (75.2%). Despite the fact that 60.6% of mothers initiate breastfeeding and 26.1% of mothers are found to breastfeed up to 2 years, the practice of EBF for the first 6 months is low (17.9%). Factors found to influence infant feeding practices are type of delivery, parity, alcohol consumption, occupation, education, and breast problems.

## 1. Introduction

Adequate nutrition during infancy and early childhood is essential to ensure the growth, health, and development of children to their full potential [1]. It has been recognized worldwide that breastfeeding is beneficial for both the mother and child, as breast milk is considered the best source of nutrition for an infant [2]. 

The World Health Organization (WHO) recommends that infants be exclusively breastfed for the first six months, followed by breastfeeding along with complementary foods for up to two years of age or beyond [3]. Exclusive breastfeeding can be defined as a practice whereby the infants receive only breast milk and not even water, other liquids, tea, herbal preparations, or food during the first six months of life, with the exception of vitamins, mineral supplements, or medicines [4]. The major advantage of exclusive breastfeeding from 4 to 6 months includes reduced morbidity due to gastrointestinal infection [5]. However, many researchers are questioning if there is sufficient evidence to confidently recommend exclusive breastfeeding for 6 months for infants in developed countries due to the fact that breast milk may not meet the full energy requirements of the average infant at 6 months of age [6]. Nevertheless, there is scanty data that give estimation about the proportion of exclusively breastfed infants at risk of specific nutritional deficiencies. 

Several studies have shown that mothers find it difficult to meet personal goals and to adhere to the expert recommendations for continued and exclusive breastfeeding despite increased rate of initiation [7]. Some of the major factors that affect exclusivity and duration of breastfeeding include breast problems such as sore nipples or mother's perceptions that she is producing inadequate milk [4, 8, 9]; societal barriers such as employment and length of maternity leave [9]; inadequate breastfeeding knowledge [8]; lack of familial and societal support; lack of guidance and encouragement from health care professionals [2, 9]. These factors in turn promote the early use of breast milk substitute. 

When breast milk or infant formula no longer supplies infants with required energy and nutrients to sustain normal growth and optimal health and development, complementary feeding should be introduced [10]. According to the WHO recommendations, the appropriate age at which solids should be introduced is around 6 months [11] owing to the immaturity of the gastrointestinal tract and the renal system as well as on the neurophysiological status of the infant [12]. Factors that influence the weaning process include infant feeding problems such as refusal to eat, colic, and vomiting among others [13]. These factors represent challenges for mothers and in turn may either directly or indirectly influence the feeding pattern. Hence, understanding the factors affecting infant nutrition in Mauritius can help in developing strategies to promote breastfeeding and overcoming problems faced by mothers and children. 

Predictors of breastfeeding and weaning practices vary between and within countries. Urban or rural difference, age, breast problems, societal barriers, insufficient support from family, knowledge about good breastfeeding practices, mode of delivery, health system practices, and community beliefs have all been found to influence breastfeeding in different areas of developing countries [4, 8, 9]. Information on the prevalence and factors influencing infant feeding practices is limited in Mauritius and dates back to 1996 [14]. This present study aims to determine infant feeding pattern and its predictors among Mauritian mothers with the following objectives: (1) to elucidate breastfeeding practices, in terms of initiation, exclusivity, and termination, and the factors influencing them; (2) to determine the time when weaning starts, the challenges met by mothers, and the type of weaning adopted.

## 2. Methods

### 2.1. Study Design and Data Collection

A survey-based study was conducted on a group of 500 mothers in 2011 (from August 2011 to January 2012) to elicit information about infant feeding practices by the use of a properly designed questionnaire given to mothers in Area Health Centres (AHCs) and Community Health Centres (CHCs) both in rural and urban areas of the island. Research has been granted approval by the University Research Ethics Committee, and prior consents were obtained from all participants.

#### 2.1.1. Questionnaire Design

The questionnaire consisted primarily of a closed format including dichotomous questions (e.g., yes/no) and multiple response for ease of completion and analysis. The resulting questionnaire consisted of 46 close-ended questions, all categorized in 4 sections as follows.

- *Section A*: the first section elicited information on the participants in terms of age, place of residence, marital status, type of family, parity, lifestyle factors (smoking and alcohol consumption), education, occupation, income, religion, and age of baby.
- *Section B*: this section was sought to understand the main factors encouraging mothers to breastfeed, their awareness on colostrum, the practice of exclusive breastfeeding, the termination of breastfeeding, as well as the main problems encountered during breastfeeding.
- *Section C*: multiple response questions were mainly used in this section to determine more information on the uptake of infant formula.
- *Section D*: it consists of dichotomous and multiple response questions to find out more details on the weaning process.

#### 2.1.2. Subjects

A sample of the female population consisting of mothers aged 18–45 years was considered since they are adults and are mature enough to participate in the study. In addition, the sampling was based on the following inclusion and exclusion criteria.

*(i) Inclusion Criteria*. Mothers who already delivered their baby and those with a child who is below 5 years old were considered in this survey.

*(ii) Exclusion Criteria.* Pregnant women or mothers having a child with any kind of malformations. Mothers with children who are above 5 years old.

### 2.2. Statistical Analysis

Questionnaire responses were collected and analysed using SPSS (version 13.0). Chi-square tests were used to evaluate relationships between different selected variables (e.g., to find association between breastfeeding initiation and mode of delivery; association between breastfeeding duration and parity, alcohol consumption, education, and occupation of respondents). The critical value for significance was set at *P* < 0.05 for all analyses.

## 3. Results

### 3.1. Breastfeeding Practices

A total of 500 respondents completed the questionnaire of which 216 were from urban areas and 284 were from rural areas, with 53% mothers having completed at least secondary level education. Equal representation of mothers from rural and urban areas was achieved through a quota sampling technique based on place of residence [15]. The age of the participants ranged from 18 to 45 years old whereby the majority of the participants (38.4%) belonged to the age group 25–31 years and most of them were married (92.6%) living in a nuclear family (58.6%). A total of 93.4% of the mothers acknowledged that they breastfed their infants of which 64.7% stated that they were self-motivated to opt for the natural way of feeding their infant since they were aware of the health benefits of breast milk and claimed that “breast milk is best.” 

### 3.2. Initiation of Breastfeeding

Additionally, 60.6% of the participants initiated breastfeeding the same day after delivery, while 39.4% started to nurse their baby 24 hours after delivery. Chi-square (*χ* ^2^) test confirmed that the timing of *breastfeeding initiation* was significantly associated with *mode of delivery* (*χ* ^2^ = 212, *P* < 0.001). It should be noted that there were a greater number of mothers, that is, 294 participants (58.8%) delivered their infants by the normal vaginal method compared to 206 mothers (41.2%) who delivered by the caesarean method. It has been observed that 42.6% who had a normal vaginal delivery initiated breastfeeding immediately or within minutes after birth compared to 23.9% of those who had a caesarean type of delivery.

### 3.3. The Practice of Exclusive Breastfeeding

Although 35.7% of the participants had adequate knowledge on the definition/meaning of EBF, the practice was relatively low compared to the WHO recommendation, whereby only 17.9% of the women gave their infants only breast milk during the first six months. 

The main deterrent of EBF is the early introduction of water (Table 1) and infant formula (Table 3). It is worth noting that mothers stated during the survey that they started to give water around 2 months. Other major barriers to EBF include employment (27.3%) followed by milk insufficiency (22.6%) as reported by the respondents. 

**Figure 1:**
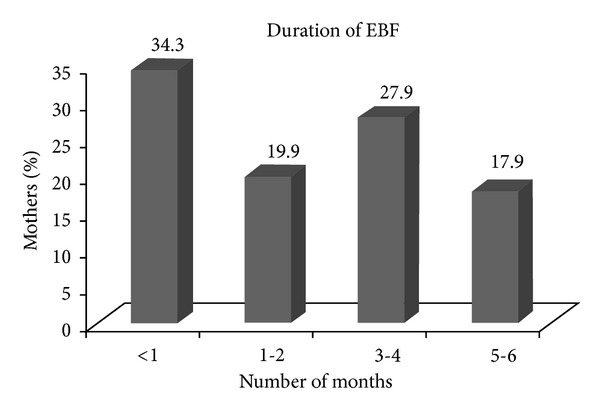
Duration of exclusive breastfeeding.

These factors in turn led to a very short *mean duration* of EBF that is 2.10 months.  Figure 1 depicts the number of months that mothers have exclusively breastfed their infants. The majority of the women practiced exclusive breastfeeding for less than one month (34.3%), while only 17.9% of them breastfed their child exclusively for around 5-6 months. 

### 3.4. Factors Influencing Breastfeeding Duration

The majority of the mothers completely terminate breastfeeding around 19–24 months (26.0%); *χ* ^2^ test confirms that there are associations between the *duration of breastfeeding* and *parity, alcohol consumption, education*, and *occupation* of the respondents, while *age group, residence, type of family*,  and *type of delivery* were not statistically significant (*P* > 0.05). These data are shown in Table 2.

It has been found that more primiparous mothers would stop nursing their infants around 19–24 months (34.5%) compared to multiparous mothers (19.9%), and cessation of breastfeeding beyond 24 months is more prevalent among participants who never drink alcoholic beverages. As far as education is concerned, it has been seen that irrespective of the level of schooling attained, mothers usually stop breastfeeding their infants within 24 months. In addition, even if women are employed as professionals (28.3%) or are housewives (26.3%), they are more likely to discontinue breastfeeding within 24 months. 

**Figure 2:**
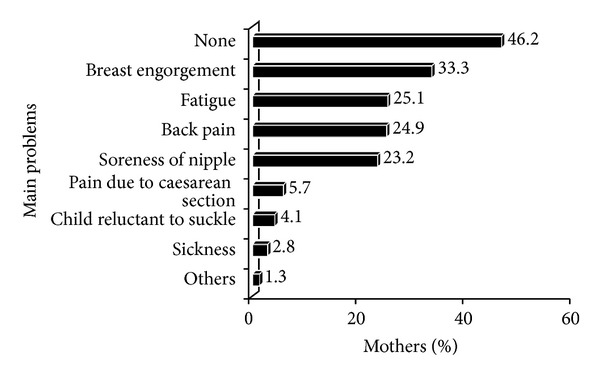
Main problems encountered during the breastfeeding process.

During the breastfeeding process, many mothers complained about the problems they encountered. It can be seen from Figure 2 that the majority of the respondents (46.2%) did not face any problems while breastfeeding, but among those having difficulties, breast engorgement was most prevalent (33.3%) followed by fatigue (25.1%), back pain (24.9%), and soreness of nipple (23.2%), while pain due to caesarian section, reluctance of infant to suckle, or sickness were minor problems that mothers faced.

With respect to the introduction and use of infant formula, results indicate that more participants (37.9%) start to use breast milk substitute within one month after delivery, whereby 33.9% of participants who use infant formula highlighted milk insufficiency as being the major reason to bottle feed, while 32.5% reported that they had to resume work; thus, they opted for formula feeding as shown in Table 3.

Though the majority of the mothers reported that they did not have any problems with the breast milk substitute, that is, they never had to change the type of formula milk used (80.8%), some reported that baby constipated (5.9%) and fell sick (4.3%) with the infant formula, respectively. 

### 3.5. Weaning Introduction

Complementary feeding was more commonly initiated around 4–6 months (75.2%) and partial weaning (when baby is breastfed once or twice per day while receiving complementary foods) was the most common type of weaning practiced by mothers (62.8%). During complementary feeding, both home-made and commercially available foods (cereals, ready-made pots) are given to the infants (69.2%). It has been found that weaning started with mashed vegetables or fruits (66.9%) and the main reasons are due to the freshness of home-made food and it is also more hygienic (93.5%). Additionally, 86.4% of the participants reported that the nutritional quality of home-made food is superior to that of commercial food, while 84.9% of the women stated that food prepared at home provides room for more choices for a balanced meal. With regard to the commercially available baby foods, it was noted that mothers prefer cereals (34.1%) to ready-made pots (7.80%). It has also been found that 68.7% of mothers did not encounter any difficulty with their infants during the weaning period. Moreover, the other respondents (21.7%) highlighted that their children were unwilling to take solid foods, while 19.6% of them reported that their infants prefer drinking to food. 

## 4. Discussion

A higher standard of living coupled with a higher education level in Mauritius during the last 20 years has resulted in more women in the working sector. However, this has not dramatically decreased breastfeeding practice as it has been noted that the prevalence of breastfeeding in Mauritius has risen from 72% in 1991 [14] to 93.4% as found in this study. This may reflect the success of health promotion campaigns reiterating that “breast is best” or “breast milk is beneficial for babies and mothers” which account for the fact that mothers were self-motivated to breastfeed. Findings of this study are consistent with the one conducted in Northern Ireland [16] which reported that mothers were encouraged to breastfeed only because they know that “breast is best” or owing to the benefits of breast milk. 

### 4.1. Initiation of Breastfeeding

Although WHO's, Global and National Infant, and Young Child Feeding Guidelines recommend that all newborns should start breastfeeding immediately (within the first hour after delivery), the current study showed that very few participants (27.2%) started to breastfeed immediately/within minutes after delivery or within one hour after birth compared to 39.4% mothers who initiated breastfeeding later than 1 hour within the same day. Additionally, caesarian delivery in Mauritius is on the rise. It has been noted that 206 respondents delivered by caesarian section of which 76.1% began to breastfeed their infants after 24 hours of birth. The delayed initiation of breastfeeding was most probably related to (1) the physical condition of the mother after delivery [17], whereby some mothers claimed that they were not feeling well enough to be able to breastfeed; (2) painful conditions associated with caesarian section; (3) the absence of their infants who were kept in nursery. 

Similarly, other studies also noted that the rate of breastfeeding initiation within 1 hour was low and the principal barrier to the initiation and even continuation of breastfeeding is due to the operative obstetrical intervention [17–19]. It has also been reported that after the caesarean section, mothers and infants are separated for a long period of time owing to anesthesia, baby being kept in nursery, or mother being sedated for pain and unable to feed [19, 20]. This ultimately leads to poor maternal milk surge. 

### 4.2. Exclusive Breastfeeding

It has been found that although knowledge on EBF for the first 6 months as per WHO recommendation (35.7%) was relatively high, only about half (17.9%) actually practiced it. The mean duration of exclusive breastfeeding in Mauritius is only 2.10 months, whereby there are 17.9% of mothers who practiced EBF for the first 6 months unlike in other developing countries such as East Asia/Pacific which have the highest rate of exclusive breastfeeding (43.0%) followed by Eastern/Southern Africa (41.0%) (UNICEF, the United Nations Children's Fund) [21]. Therefore, it can be argued that mothers failed to adhere strictly to the WHO recommendation of EBF for the first 6 months owing to the introduction of water and infant formula much before 6 months. 

The main determinants of EBF include resumption of work followed by milk insufficiency. Usually, female workers in Mauritius are allowed 12 weeks of maternity leave which equals to approximately 3 months (SSPTW, Social Security Programs Throughout The World) [22]. Under these circumstances, mothers are prompted to resort to the supplementation of infant formula before 3 months so that their infants familiarize to bottle feeding during their absence. This finding is consistent with other studies which highlighted employment and milk insufficiency as the major barriers to EBF [2, 8, 23–25], while another research pointed out that mothers stop EBF as they perceive that their infants feel hungry and unsatisfied with breast milk only [8]. They ultimately resort to supplement with infant formula. 

Nevertheless, it has been argued that the exclusivity of breastfeeding is affected when mothers experience problems with the infant latching-on or sucking and they do not get assistance from some clinicians who do not feel intrepid in their skills to support breastfeeding and may have limited time to address the matter during preventive visits [26]. Additionally, 26.0% of the respondents cease breastfeeding within 2 years, while there are notably some mothers who breastfeed above 2 years. This implies that despite the fact that the majority of the participants adopt mixed feeding, they still adhere to the WHO recommendation which involves continued breastfeeding up to 2 years or beyond [1]. This study reveals that factors including *parity, alcohol consumption, education,* and *occupation* are associated with the termination of breastfeeding.

### 4.3. Factors Associated with the Duration of Breastfeeding

#### 4.3.1. Parity and Alcohol Consumption

There are a greater number of women from the lower parity who terminate breastfeeding within 2 years as compared to their counterparts. Primiparous women are less knowledgeable and skilful in breastfeeding [25]; hence, they will usually seek assistance, advice, and help from health care professionals who generally promote breastfeeding. Furthermore, first time mothers are more likely to consider health promotion messages or be exposed to them in different ways [27]. On the other side, higher parity leads to short birth intervals, hence, minimal time available for breastfeeding [28]. In contrast to previous reviews, primiparity was associated with reduced risk for breastfeeding duration [29], while other studies done in the United Kingdom [30] and in Bangladesh [31] affirmed that breastfeeding duration increases with increasing parity which might be related to previous breastfeeding experiences. Nevertheless, in another study [32], it was asserted that parity had no significant influence on duration of breastfeeding.

There is an association between the frequency of intake of alcoholic beverages and duration of breastfeeding. Mothers who never or seldom take alcoholic drinks are more apt to breastfeed longer than those who consume them occasionally. This might be because those who consume alcohol on a regular basis avoid breastfeeding owing to the fact that alcohol readily crosses into breast milk by simple diffusion, attaining levels approximately equal to that in the maternal blood stream [33]. 

This finding agrees with those observed in other studies carried out in Australia [34] and in Greece [35] which stated that mothers stop breastfeeding their infants earlier because exposing the child to small amounts of alcohol through breast milk disrupts infant sleeping patterns.

#### 4.3.2. Education and Occupation

It was noted that the level of education did not have any influence on breastfeeding duration and Mauritian mothers usually breastfeed at least for 12 months.

In contrast to this study, it has been found in a previous research conducted in Philippines [28] that education plays a significant role in determining the duration of breastfeeding. Increasing level of education also implies adoption of modern ideas while gradually leading to the dereliction of traditional practices regarding child care, thus, a decrease in the rate of breastfeeding.

With regard to occupation of the mothers, it has been observed that regardless of the fact that the participants are housewives or employed as professionals, they would normally stop nursing their infants within 2 years. Generally, housewives have unlimited time available to feed their infants while on the other hand, despite the fact that the participants work as professionals, they still breastfeed as long as housewives do. One most probable reason for this is that even though they work, they express their breast milk either manually or with pumps so that somebody else can still feed the baby or they are usually given flexible time at work to maintain breastfeeding. Another study in Malaysia [36] reported that facility at workplace similar in Mauritius such as allowing mothers a flexible time to express breast milk helps in maintaining lactation. This issue of breast milk expression needs to be addressed in future studies. Conversely, other investigators observed that women having professional jobs especially in urban areas stop breastfeeding earlier than the recommended duration because they have reduced access to their children whereas those involved in traditional work have more time and maintain longer periods of lactation [28]. 

### 4.4. Breastfeeding Challenges

Although a greater part of the participants do not experience any difficulties while nursing their infants, there were still a significant number of the respondents who complain about breast engorgement, fatigue, back pain, and soreness of nipple. Breast engorgement usually occurs when milk gets accumulated in the breast, while sore nipples arise because of the baby sucking the nipple area of the breast only [37]. Generally, nursing mothers breastfeed their children frequently during the day (each 2 hours) which leads to fatigue and back pain. This research affirmed that these difficulties result in a negative experience with breastfeeding which is followed by a decrease in mothers' confidence to wet-nurse their infants, hence, causing early cessation of breastfeeding [38]. These results are consistent with recent studies demonstrating that many women encounter problems such as cracked nipples, low milk supply, and breast engorgement [24, 26, 37–41]. 

### 4.5. Infant Formula Feeding

Early termination of breastfeeding also implies early use of breast milk substitute and as pointed out above, factors such as work, milk insufficiency, and breastfeeding difficulties are the major reasons for adopting formula feeding. Among the few participants who encountered minor feeding problems with the formula milk reported constipation and sickness such as vomiting, diarrhoea, colic, and regurgitation as the most common ones. The risk of constipation among formula-fed children is quite common and this has also been found in Italy [42], whereby the authors reported that there is a prolonged gastrointestinal transit in formula-fed infants and the stool consistency is hard compared to breastfed infants. 

### 4.6. Weaning Introduction

Complementary foods are generally introduced between 4 and 6 months and partial weaning is the most common type of weaning adopted by mothers. Generally, women who terminate breastfeeding within 2 years are more likely to adopt partial weaning because it involves nursing the infant as well as introducing complementary foods [43], while those who stop nursing their infants within 6 months adopt mother-led weaning. Conversely, mother-led weaning occurs when the mother feels the need to introduce complementary foods. Since, there is limited research on the type of weaning adopted by mothers during infant feeding practices, the results obtained in the present study are more suggestive than affirmative.

Results herein corroborate those carried out in Switzerland [12] which demonstrate that a greater number of women start to wean their infants with mashed vegetables or fruits followed by cereals. The main reason as pointed out by the participants in this study is that home-made food is more fresh, nutritious, and hygienic unlike commercially available cereal or baby foods. Gradually, baby cereals or commercial purees are also used alongside home-made foods and more women prefer cereals (34.1%) to ready-made pots (7.80%) because they believe that commercial purees contain additives, high sugar content and salt content, compared to cereals. To date, there are no published data on the type of weaning food (home-made versus commercially available food). 

A few mothers experience difficulties during complementary feeding which include unwillingness of the child to eat while exerting preferences to drink rather than eating. The minority of the participants affirmed that they encountered problems such as allergic reactions and health problems with the infant including vomiting, colic, and diarrhoea which may arise due to the feeding practices adopted by mothers [44]. Other possible barriers during complementary feeding found in other studies unlike the present study include food refusal, selective, picky or fussy eating, eating slowly, being less interested in food, and having a small appetite [44]. 

## 5. Conclusion and Limitations

This study shows that the prevalence of breastfeeding has increased over the past 20 years in Mauritius. The WHO guidelines advise to breastfeed exclusively until 6 months of age. Despite a high breastfeeding initiation rate of 61%, only 18% succeed to give exclusive breastfeeding until 5-6 months. The mean duration of exclusive breastfeeding is 2 months, with adding water as the main reason for not continuing exclusiveness. Awareness of the health benefits of breastfeeding was noted in 65%, a percentage that may be increased by further breastfeeding education and support. The major barriers to breastfeeding practices in this study in terms of initiation, exclusivity, and duration are (1) type of delivery; (2) parity; (3) alcohol consumption; (4) occupation and education; (5) breast problems, mainly milk insufficiency. 

These factors encourage early use of formula milk. On the other hand, complementary foods are normally introduced around 4 to 6 months and mothers usually start with home-made food because of its freshness and for hygienic reasons. However, there are very few mothers who encountered difficulties during the weaning process as compared during breastfeeding practices such as refusal to eat followed by vomiting, colic, allergic reactions, and diarrhea which were rare.

There are two major limitations in our study. Future studies along the same line should target children of 3 years as it has been suggested by Khassawneh that this will reduce the risk of recall bias [45]. 

To calculate the sampling size, the female population in the reproductive ages was considered. However, this data is not representative of the number of mothers aged between 18 and 45 years.

## Figures and Tables

**Table 1:** Reasons for not adhering to the WHO recommendations of exclusive breastfeeding for the first six months.

Reasons	Frequency	
	*n*	%
Introduction of water	116	30.1
Resumption of work	105	27.3
Milk insufficiency	87	22.6
Mother's desire	50	13.0
Baby too demanding/not satisfied	42	10.9
Unwillingness of the child to suckle	37	9.6
Medical complications	15	3.9
Had to take medication	11	2.9
Lack of time	5	1.3
Too painful	3	0.8

**Table 2:** Link between cessation of breastfeeding and socioeconomic and demographic factors. Termination of breastfeeding in months.

Category	<1		1–6		7–12		13–18		19–24		25–30		31–36		>36		Results of chi-square test	
	*n*	%	*N*	%	*N*	%	*n*	%	*n*	%	*n*	%	*n*	%	*n*	%	*χ* ^2^ value	*P* value
Age																		
18–24	2	14.3	25	25.3	23	29.9	11	32.4	41	33.6	8	25.0	18	28.1	3	10.7	25.7	0.220
25–31	7	50.0	37	37.4	24	31.2	0	29.4	55	45.1	15	46.9	19	29.7	13	46.4		
32–38	3	21.4	23	23.2	19	24.7	8	23.5	17	13.9	5	15.6	21	32.8	6	21.4		
39–45	2	14.3	14	14.1	11	14.3	5	14.7	9	7.4	4	12.5	6	9.4	6	21.4		
Residence																		
Rural	4	28.6	41	41.4	36	46.8	14	41.2	57	46.7	7	21.9	28	43.8	12	42.9	8.26	0.310
Urban	10	71.4	58	58.6	41	53.2	20	58.8	65	53.3	25	78.1	36	56.3	16	57.1		
Parity																		
Primiparous	6	42.9	44	44.4	34	44.2	12	35.3	67	54.9	9	28.1	15	23.4	7	25.0	24.3	**<0.01**
Multiparous	8	57.1	55	55.6	43	55.8	22	64.7	55	45.1	23	71.9	49	76.6	21	75.0		
Type of family																		
Nuclear	9	64.3	55	55.6	44	57.1	22	64.7	66	54.1	22	68.8	37	57.8	17	60.7	3.48	0.837
Extended	5	35.7	44	44.4	33	42.9	12	35.3	56	45.9	10	31.3	27	42.2	11	39.3		
Type of delivery																		
Normal vaginal	7	50.0	66	66.7	42	54.5	23	67.6	65	53.3	19	59.4	33	51.6	17	60.7	11.6	0.638
Caesarian section	7	50.0	32	32.3	35	45.5	11	32.4	57	46.7	3	40.6	31	48.4	11	39.3		
Ventouse	0	0.0	1	1.0	0	0.0	0	0.0	0	0.0	0	0.0	0	0.0	0	0.0		
Alcohol consumption																		
Occasionally	3	21.4	37	37.4	25	32.5	13	38.2	39	32.0	7	21.9	29	45.3	10	35.7	29.5	**0.009**
Seldom	4	28.6	10	10.1	6	7.8	5	14.7	5	4.10	4	12.5	10	15.6	7	25.0		
Never	7	50.0	52	52.5	46	59.7	16	47.1	78	63.9	21	65.6	25	39.1	11	39.3		
Education level																		
Primary	1	7.1	21	21.2	12	15.6	6	17.6	22	18.0	13	40.6	18	28.1	13	46.4	46.6	**0.015**
Secondary	10	71.4	52	52.5	45	58.4	16	47.1	59	48.4	14	43.8	36	56.3	14	50.0		
HSC	2	14.3	17	17.2	12	15.6	9	26.5	18	14.8	3	9.4	5	7.8	1	3.6		
Diploma	0	0.0	1	1.0	4	5.2	1	2.9	7	5.7	0	0.0	6	0.0	0	0.0		
Graduated	1	7.1	8	8.1	4	5.2	2	5.9	16	13.1	2	6.3	5	7.8	0	0.0		
Occupation																		
Student	0	0.0	0	0.0	2	2.6	0	0.0	1	0.8	0	0.0	0	0.0	0	0.0	41.8	**0.045**
Blue collars	2	14.3	19	19.2	7	9.1	3	8.8	18	14.8	6	18.8	11	17.2	6	21.4		
White collars	3	21.4	30	30.3	21	27.3	11	32.4	34	27.9	4	12.5	13	20.3	4	14.3		
Housewife	4	28.6	42	42.4	43	55.8	18	52.9	64	52.5	20	62.5	35	54.7	17	60.7		
Self-employed	5	35.7	8	8.1	4	5.2	2	5.9	5	4.1	2	6.3	5	7.8	1	3.6		

**Table 3:** Reasons for opting infant formula.

Reasons	Frequency	
	*n*	%
Milk insufficiency	127	33.9
Resumption of work	122	32.5
Unwillingness of the child to suckle	55	14.7
Mother's desire	51	13.6
Other's	44	11.7
Medical complications	29	7.7
Doctor's recommendation	8	2.1
Aesthetic reason	1	0.3
